# Supplementary material for: Learning curve and surgical outcome of robotic assisted colorectal surgery with ERAS program
Source: Sci Rep. 2022 Nov 29;12:20566. doi: 10.1038/s41598-022-24665-w (PMC9709162; doi:10.1038/s41598-022-24665-w)
Supplement: Supplementary file 1 — Supplementary Information 1. [file 41598_2022_24665_MOESM1_ESM.zip › LSC Raw data-Ñ[▒K/085/H_ñw╝╨░O▒Kñσ.pdf]

正式摘要(電子簽章：000000196274205)

W55 / W55 ☐ 英文標題(注意:無英文姓名-請補病人基本檔!) ☐ 【讀取】憑證

臺中榮民總醫院

出院病歷摘要

轉入號： 轉出號：

[redacted]

(8)流水編號	
(9)入院日期	109/07/21 (2020) 直腸外科 W55 - 252
(10)轉科日期	
(11)出院日期	109/08/07 (2020) 住院天數計 17 日

診斷

- 入院 :1. D colon adenocarcinoma 50 cm FAV s/p loop colostomy on 2020/7/6, cT3N1M0
  2. Osteonecrosis of left femoral head, Ficat stage IV, s/p left THR on 104/11/03.
- 出院 :1. Malignant tumor of distal D-colon with total obstruction, s/p loop T-colostomy, s/p 3D laparoscopic left hemicolectomy on 20200722, pT4aN0(0/27)M0, stage IIB, MD-PD, MSS, AI-, PI-, TuDp-, CRM-, ExtN-.
  2. Osteonecrosis of left femoral head, Ficat stage IV, s/p left THR on 104/11/03

主訴

- bowel obstruciton s/p loop T 2 wk ago.

病史

- This 65-year-old male with past history of
  1.D colon adenocarcinoma s/p loop colostomy on 7/6, cT3N1M0
  2.Osteonecrosis of left femoral head, Ficat stage IV, s/p left THR on 104/11/03.
  He suffered from abdominal pain for a week. He went to 員榮 and KUB showed ileus. Then he was transferred to 員生H for abdominal CT. Lab data showed AFP 2.4, CEA 3.7, CA-199 11.4, PSA 0.726. Abdominal CT on 7/6 showed colon cancer and ileus. NG with free drainage was performed. Therefore, he was transferred to our ER for operation due to colon cancer. At our ER, PE revealed abdominal diffuse tenderness. Loop colostomy was arranged on 7/6. This time, he was admitted for 2nd stage operation.

體檢發現

- MRI: SMA thrombosed.
  - General appearance :
    - easy looking , conscious clear , without cardiopulmonary distress
    - Vital signs自動帶入date 體溫(BT) 脈搏(P) 呼吸(R) 血壓(BP) 身高(BH) 體重(BW) BMI
      20200721 36℃ 91次/分 18次/分 136/91mmHg 172cm 45.9kg 15.5
    - Vital signs: B.P. 136/91 mmHg , P.R. 91 /min , R.R. 18 /min , B.T. 36 ℃
  - HEENT :
    - Eyes: no anemic conjunctiva, no icteric sclera, normal EOM
    - ENT: no otorrhea, no rhinorrhea, no erythematous mucosa, no injected tonsil, no deformity
  - Neck: supple, freely movable, trachea midline,no deformity, no engorged jugular vein , no palpable thyroid or lymph node enlargement
  - Chest:
    - Inspection: Grossly normal, symmetric and fully expansion, no accessory muscle used, no operation scar, no spider angiomata, no gynecomastia
    - Palpation: no palpable mass or nodule, no subcutaneous emphysema, no tenderness
    - Ausculation: clear breathing sound
  - Heart:
    - Percussion: normal heart size
    - Ausculation: regular heart beat, normal S1, S2, no gallop, no murmur
  - Abdomen:
    - Inspection: in
      - flat contour, symetrically, no scar, no stria, no dilated veins, no rashes
    - Ausculation: normoactive bowel sound, no abdominal bruits
    - palpation: soft, no muscle guarding or rigidity, no local tenderness, no rebounding pain, no palpable mass, no palpable pulsation
    - Percussion: no shifting dulllness, no hepatomegaly, no splenomagaly
  -

Peripheral pulsation :	CA	BA	RA	FA	PA	PTA	DPA
Right side :	++	++	++	++	++	++	++/+++
Left side :	++	++	++	++	++	++	++/+++

  - Genito-anus: no tenderness or palpable mass lesion or rectal shelf, normal external genitalia, normal anal tone, brown stool

CRS CONDITION

  - CEA: 3.7 ng/ml
  -
    - Lung

手術日期及方法

- 手術日期及方法20200722 1. 3D Laparoscopic left hemicolectomy with side-to-side anastomosis, end-GIA 60/4.1*3, isoperstalsis, re-inforcement with silk: done.
  2. Vessel ligation: left branch of middle colic and left colic vessels, S1 vessel.
  3. Cut margin: proximal 9 cm, distal 8 cm.
  4. Reperitonealization: Nil.
  5. Drain : nil.

住院治療經過

- The surgery was done on 07/22. After the operation, his vital sign was stable and no fever was noted. Vomiting with green discharge was noted on 07/24, so we prescribed NG tube with decompression. No more vomiting was noted after that. Pus discharge from the surgical wound was noted, so we wet dressing the wound from 7/27-8/6. The wound healed well and covered with granulation tissue. Duoderm gel was suggested for CD. On 7/28, we start to try diet. Now his general condition is stable with increased appetite. We will discharged him and follow him at OPD next week.

合併症

一般檢查

- 累積報告 :

DATE	TIME	ALB	TP	BIL.T	BIL.D	ALKP	AST	ALT	LDH	CHOL	TG	HDL-C	LDL	UA	NH3	AMY
1090706	1735			1.2				15								
1090708	0908			0.61		73	18	6								
1090713	0931	2.7		0.32			22	10								
1090721	1840	3.5		0.2	0.1		34	30								
1090723	0919	2.9		0.39	0.13		19	17								73

DATE	TIME	WBC	RBC	HGB	HCT	MCV	PLT	BLST	NEUT	BAND	SEG	LYM	MON	EOS	BAS
1090706	1735	5480		14.1			270		69.3			17.5	12.4	0.4	0.4
1090708	0916	4660		11.9			193		74.9			16.1	8.4	0.6	0.0
1090713	0908	6250		11.1					73.5			16.2	8.6	1.4	0.3
1090721	1841	5640	4.40	12.3	36.7	83.4	363		58.7			26.4	12.6	1.8	0.5
1090723	0911	8960	4.25	11.8	34.9	82.1	400		80.8			10.8	8.3	0.0	0.1
1090803	0911	5560	3.51	9.8	29.3	83.5	417		62.9			23.2	11.3	2.2	0.4
1090806	0905	5580	3.62	10.1	30.2	83.4	387		58.9			26.2	11.5	2.9	0.5

DATE	TIME	NA	K	CL	CA	BUN	CREAT	Mg	P	CRP	ESR	PCT	LIPASE	LACTATE
1090706	1734													24.8

1090706	1735	123	4.1			17	1.03							
1090708	0908	123	3.5	95	7.3	5	0.74							
1090708	1652	129												
1090709	0900	132												
1090713	0931	129	3.1	96	7.6	5	0.70							
1090721	1840	130	4.5	97	8.7	15	0.86							
1090723	0907												17	
1090723	0919	135	4.2		8.1	12	0.78							
1090803	0930	135	4.3			7	0.68							
1090806	0914	130	4.4			7	0.64							

DATE	TIME	GLUCOSE	PAB	TRF	ZN	CU	KETONE	CK	CKMB	PT-P	PT-C	APTT-P	APTT-C	TRO-T
1090706	1733									10.2	10.3	29.7		
1090707	0612	215												
1090721	1840									10.4	10.3	27.8		

DATE	TIME	PH	BEB	SCVO2	PO2	HCO3	HGB	PCO2	TCO2
1090706	1734	7.270	-6.3	29.7	22.9	20.6		45.9	22.0

- 放射線報告 :檢驗項目 **:CT, LUNG** 報告日期 **:1090715**

主　訴：
Result:
Finding:
CT of chest and mediastinum with IV contrast enhancement:
> Colon Ca. s/p operation for lung survey.
> A tiny calcified nodule over left major fissure(axial:38), probably benign in nature.
> No CT evidence of lung metastasis.
> Marked emphysematous change over bilateral lungs, COPD should be considered.
> No obvious enlarged LNs in the mediastinum.
> A 0.9cm hepatic cyst at lateral segment of liver.
> Multiple tiny low attenuated lesions in both lobes of liver, too small to determine nature.
> Relative prominenct gallbladder wall thickening and enhancement, Suggest clinical correlation to R/O cholecystitis.
Impression:
1. No CT evidence of lung metastasis.
2. Relative prominenct gallbladder wall thickening and enhancement, Suggest clinical correlation to R/O cholecystitis.

檢驗項目 **:MRI,LIVER** 報告日期 **:1090716**

主　訴：
Result:
Finding:
MRI of liver pre and post iv contrast:
1. Axial & Cor. HASTE T2WI without fat-saturation
2. Axial in- and out-phase GRE phase contrast MRI
3. Axial thin-slice dynamic MRI of liver pre and post bolus
iv injection of Gd-contrast agent.
4. Cor. post contrast fat-saturated GRE T1WI
5. Axial diffusion weighted imaging (DWI) of liver
> A case of colon cancer s/p operation with ostomy at right lower abdomen. For liver lesions survey
> Multiple liver cysts upto 0.9cm in size are noted at bilateral lobes of liver
> Several wedged lesions with early enhancement in arterial phase and isoenhancement in delayed phase in liver parenchyma near the gallbladder fossa are noted. The lesions might be transient hepatic signal intensity differences (THAD).
> Filling defect with proximal SMA(Ser:15, Img:29) is noted, maybe related to SMA thrombosis. The distal SMA shows patent blood supply.
Impression:
1. A case of colon cancer s/p operation with ostomy at right lower abdomen. No obvious liver cirrhosis is noted
2. R/O proximal SMA thrombosis

- 檢驗項目 **:PET WHOLE BODY SCAN** 報告日期 **:1090717**

```
*  Finding:                                              *
*  The PET/CT scan with F-18 FDG from skull base to upper thigh:     *
*  .                                                     *
*  1. A huge mass at the descending colon (SUVmax:17.8/1h).        *
*  2. A small FDG-avid lesion at the left border of rectrum (SUVmax   *
*  3. Mildly increased FDG uptake at the bilateral pulmonary hilar    *
*  4. Mildly increased FDG uptake at the right lower lung (SUVmax:1   *
*  5. No abnormal tracer uptake is demonstrated elsewhere in this s   *
*  .                                                     *
*  Impression:                                           *
*  1. The huge mass at the descending colon may be compatible with   *
*  2. The small FDG-avid lesion at the left border of rectrum may b   *
*  3. The mildly increased FDG uptake at the bilateral pulmonary hi   *
*  4. No definite evidence of FDG-avid tumor is demonstrated elsewh   *
*  .                                                     *
*  * The tracer accumulation was classified into five grades:        *
*  0: benign            3: probably malignant                 *
*  1: probably benign        4: malignant                     *
*  2: equivocal                                          *
```

特殊檢查

病理報告

- 活體組織切片病理報告　手術日期：**109/07/22**　病理號：**S0921723**

診斷醫師:吳致瑩 報告日期:109/07/29
Pathologic diagnosis:
1. Desending colon, laparoscopic anterior resection --- a. Adenocarcinoma, moderately to poorly differentiated, arising from background of tubulovillous adenoma with micropapillary feature. Tumor invaded through serosa with marked tortuosity. The bilateral cut ends and mesocolic lymph nodes (0/24) are free of tumor. b. Tubular adenoma with low grade dysplasia, 2 foci.
2. Left colic root, dissection --- Unremarkable lymph nodes (0/3) and fibrovasculoadipose tissue.
Ancillary study for diagnosis:
IHC stains for PMS2 and MSH6 are done on section H. Both markers are preserved.
Prognostic and predictive factor:
1. Tumor size: 8x5.5 cm.
2. Depth of invasion: Tumor invaded through the serosa with marked tortuosity.
3. Histologic grade: G2: Moderately differentiated to G3: Poorly differentiated.
4. Angiolymphatic invasion: Not identified.
5. Perineural invasion: Not identified.
6. Tumor deposits (discontinuous extramural extension): Not identified.
7. Macroscopic tumor perforation: Not identified.
8. Surgical Margins:
a) Proximal Margin: Uninvolved by invasive carcinoma, distance of tumor from margin: 7.5 cm.
b) Distal Margin: Uninvolved by invasive carcinoma, distance of

- tumor from margin: 10 cm.
  c) Radial or Mesenteric Margin: Uninvolved by invasive carcinoma, distance of tumor from margin: 2.5 cm.
  9. Lymph node status: Uninvolved (0/27).
  10. Extranodal involvement: Not identified.
  11. Tumor regression grading S/P CCRT: No known presurgical therapy.
  12. Type of polyp in which invasive carcinoma arose: Not identified.
  13. Additional pathologic findings: Adenomas.
  14. Pathological TNM Stage: pT4aN0 (According to the eighth edition, American Joint Committee on Cancer Staging Guidelines for Tumors).
  15. TNM descriptors: Not applicable.
  Gross description:
  The specimen consists of 1) a segment of colon, 17.5 cm in length and 4.5 cm in proximal cut end circumference, 4.6 cm in distal circumference, and mesocolic soft tissue. A gray tan firm annular elevated and centrally ulcerative tumor, 8x5x5.5 cm, is noted, 10 cm to the distal cut end. On cross section, the tumor invades through the serosa with inflammatory tract connecting to another part of mucosa (within the same segment), 2.5 cm to the radial margin, and serosal retraction is also noted. Two polyps, up to 0.8x0.7 cm, are also seen distal to the main tumor. The both cut ends are free of tumor grossly. 2) Left colic root, a piece of tan yellow soft tissue, 5.4x3.9x0.9 cm. Representative part for section: A) proximal cut end B) distal cut end C) polyps D-H, Q-T) tumor (Q-S: inflammatory tract through serosa, Q-R: mapped) J) radial margin K) omentum L) specimen 2 M-P) lymph nodes.
  Note:
  1. The interpretation of MMR panel and recommended studies are as below: No loss of nuclear expression of MMR proteins: low probability of microsatellite instability-high (MSI-H). Please note: There are exceptions to the above IHC interpretations. These results should not be considered in isolation, and clinical correlation with genetic counseling is recommended to assess the need for germline testing.
  2. This case has been peer reviewed by two doctors.
  #T-59461_2 #M-81403_2 3 1105 S0000F
  #T-C4560_2 #M-09183_2 0 1105 000000

其它

出院時情況

- 3.改門診治療

出院指示

出院計劃

- 門診日期
  - date on 2020/08/11

主治醫師_2222H 林俊余 / 住院醫師_9790D 蘇仁惠

正式摘要(電子簽章：000000196274205)

W55 / W55

doctorRank:B
